# Perceived stress mediates the relationship between professional role and trait anxiety in coaches and referees

**DOI:** 10.3389/fpsyg.2026.1837147

**Published:** 2026-07-27

**Authors:** Donatella Di Corrado, Andrea Buscemi, Paolo Arculeo, Sandra Prinzi, Maria Chiara Parisi, Marinella Coco

**Affiliations:** 1 Department of Sport Sciences, Kore University, Enna, Italy; 2 Department of Research, Centre Studies of Osteopathy, University of Catania, Catania, Italy; 3 Department of Biomedical and Biotechnological Sciences, University of Catania, Catania, Italy

**Keywords:** anxiety, coaches, personality, referees, stress

## Abstract

**Introduction:**

Understanding the psychological processes underlying anxiety in sport professionals represents a central issue in sport psychology. Although previous research has documented differences in stress and anxiety across sport roles, less attention has been devoted to examining the potential explanatory pathways through which these differences emerge, particularly within specific sport contexts such as football. The present study aimed to examine whether perceived stress mediates the relationship between professional role and trait anxiety in football coaches and referees.

**Methods:**

A total of 177 football professionals (94 referees and 83 coaches) competing at the regional level completed the State–Trait Anxiety Inventory, a short version of the Big Five personality questionnaire, and the Psychological Stress Measure. Group differences, correlations, hierarchical regression analyses, and mediation analysis were conducted.

**Results:**

Data showed that referees reported significantly higher levels of perceived stress and trait anxiety compared to coaches, whereas coaches scored higher on openness, agreeableness, and conscientiousness. Perceived stress was positively associated with both state and trait anxiety and emerged as the strongest predictor in regression analyses. Mediation analysis revealed that perceived stress significantly mediated the relationship between professional role and trait anxiety. The indirect effect was significant, while the direct effect remained significant, indicating a partial mediation.

**Discussion:**

These findings suggest that differences in anxiety between sport professionals are not solely related to role-specific demands, but also to how these demands are perceived and cognitively appraised. Perceived stress appears to represent a potential explanatory pathway associated with anxiety in sport contexts. From a theory-informed explanatory perspective, the results highlight the importance of considering cognitive appraisal processes in understanding emotional responses in high-pressure sport roles, with relevant implications for both theory and applied practice.

## Introduction

Understanding the psychological processes underlying anxiety in sport professionals represents a central issue in sport psychology. Because the psychological demands experienced by sport professionals vary across sports, findings regarding stress and anxiety cannot be readily generalized from one sporting context to another. For this reason, the present study focuses specifically on football coaches and referees. While previous research has extensively documented the presence of stress and anxiety in sport contexts, less attention has been devoted to identifying the processes through which different sport roles may lead to distinct psychological outcomes (Di Corrado et al., 2021; Ford et al., 2017; Di Corrado et al., 2025).

Sport environments are inherently demanding, requiring individuals to cope with performance pressure, evaluation, and uncertainty. Within this context, both coaches and referees operate under conditions that can elicit significant psychological strain. However, these roles differ substantially in their structural and social demands (Samuel, 2020).

Sport referees, in particular, operate in highly demanding environments that require rapid decision-making, continuous attention, and the management of complex and dynamic situations. Their role involves evaluating game actions, enforcing rules, maintaining control, and resolving conflicts, often under time pressure and in the presence of multiple stakeholders such as players, coaches, and spectators (Wolfson and Neave, 2007). Due to the subjective and highly visible nature of their decisions, referees are frequently exposed to criticism from players, coaches, fans, and the media (Pizzera et al., 2022).

These conditions contribute to elevated levels of stress and anxiety, which have been shown to negatively affect performance, well-being, and retention in officiating roles (Vargas et al., 2025).

In contrast, coaches typically operate within a different set of demands, involving long-term planning, leadership, and athlete development (Fletcher and Scott, 2010).

These differences suggest that psychological outcomes may vary as a function of role-specific demands and associated stress processes. Anxiety, a key construct in sport psychology, is commonly conceptualized as comprising both state and trait components. It is commonly defined as an unpleasant psychological state arising in response to perceived stress associated with performing under pressure (Spielberger, 1983). Anxiety can manifest either as a relatively stable personality characteristic, known as trait anxiety, or as a temporary, situation-specific condition referred to as state anxiety. Elevated levels of anxiety have been associated with impaired performance, reduced decision-making accuracy, and decreased well-being (Ong and Chua, 2021). However, the presence of anxiety alone does not fully explain why individuals in different roles may experience it to varying degrees. From a theoretical perspective, anxiety is not determined solely by objective demands, but by how individuals cognitively appraise those demands. Anxiety consists of both cognitive and somatic components and reflects a complex interaction of psychological and physiological processes. These processes suggest that anxiety should be understood as a response to perceived stress rather than objective task demands (Ford et al., 2017).

From this perspective, perceived stress emerges as a critical construct. According to transactional models of stress, the adaptation process involves not only the presence of stressors, but also individuals’ cognitive evaluation of those stressors and the resulting emotional responses (Biggs et al., 2017).

Perceived stress reflects the extent to which situations are appraised as unpredictable, uncontrollable, and overwhelming, and has been consistently associated with anxiety and psychological distress. The present conceptual model was guided by transactional theories of stress, which emphasize cognitive appraisal as a process associated with emotional responses. Nevertheless, trait anxiety is generally regarded as a relatively stable dispositional characteristic. Therefore, the proposed ordering of perceived stress and trait anxiety should be understood as theory-driven rather than as evidence of temporal precedence. Importantly, perceived stress may represent a potential explanatory pathway linking contextual demands to emotional outcomes (Phillips, 2020). In this sense, differences between sport roles may not directly lead to differences in anxiety, but may operate through variations in how stressful those roles are perceived to be. This perspective is consistent with recent approaches emphasizing the central role of cognitive appraisal processes in sport-related stress (Nogueira et al., 2025; Gomes et al., 2022). Sport contexts expose individuals to multiple stressors; however, it is not the stressors themselves that determine psychological outcomes, but rather how they are cognitively appraised. Previous research suggests that the relationship between stressors and emotional responses operates through appraisal processes, whereby individuals who perceive situations as a challenge tend to experience more positive emotions, whereas those who perceive them as a threat tend to experience more negative emotional responses (Noteboom et al., 2001; Sullivan et al., 2024).

In addition to contextual factors, personality traits may also shape individuals’ responses to stress. The Five-Factor Model (McCrae and John, 1992) provides a useful framework for understanding individual differences. Traits such as conscientiousness and agreeableness have been associated with more adaptive coping and lower levels of anxiety, whereas emotional instability has been linked to higher vulnerability to stress (Soto and Jackson, 2013).

However, personality alone is unlikely to fully account for role-related differences in anxiety, highlighting the need to consider interactions between dispositional and situational factors (Wilt et al., 2011).

Despite the relevance of these constructs, few studies have simultaneously examined professional role, perceived stress, personality, and anxiety within a unified framework. In particular, the potential mediating role of perceived stress in explaining differences between coaches and referees remains underexplored. Recent research has increasingly focused on psychological mechanisms underlying stress in sport officials. For example, mediation models have been used to explain how psychological variables influence stress responses and related outcomes in referees (Costa et al., 2026).

Moreover, stress in sport officials has been linked to multiple situational and interpersonal demands, as well as to negative psychological outcomes such as anxiety and reduced performance (Vargas et al., 2025).

Despite the growing theoretical understanding of stress appraisal and anxiety in sport psychology, the translation of this knowledge into practical strategies for coaches and referees remains limited. Most previous studies have primarily described differences in stress and anxiety across professional roles without identifying the psychological mechanisms that could inform targeted interventions. Consequently, practitioners often lack evidence-based guidance for reducing anxiety by addressing the underlying cognitive processes. Examining perceived stress as a potential explanatory pathway may help bridge this gap between theory and practice by providing a stronger theoretical basis for the development of psychological interventions aimed at improving the well-being and performance of football coaches and referees.

The present study extends previous research by adopting a theory-informed explanatory framework to examine anxiety in football professionals, specifically coaches and referees. Rather than proposing a new theoretical model, the present study provides empirical support for a theory-informed explanatory framework by examining whether perceived stress represents a potential explanatory pathway linking professional role and trait anxiety in football coaches and referees. In doing so, it applies transactional models of stress within a specific professional sport context and integrates professional role, personality traits, perceived stress, and anxiety within a single analytical framework. Football was selected because it is characterized by high cognitive, emotional, and social demands placed on both coaches and referees, making it an appropriate context in which to examine the proposed explanatory framework. Moreover, previous research suggests that perceived stress and anxiety may vary across sports because of sport-specific performance demands.

Therefore, the present study examined whether perceived stress represents a potential explanatory pathway linking professional role and trait anxiety in football coaches and referees, rather than merely describing differences between professional roles.

**Figure 1:**
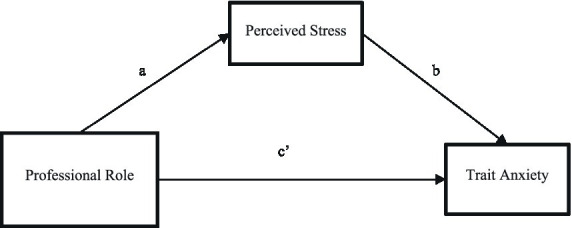
Hypothesized mediation model explaining the potential mediating effect of perceived stress on the relationship between professional role and trait anxiety.

Specifically, in the context of football, the aims of the study were: (1) to examine differences between coaches and referees in personality traits, perceived stress, and anxiety; (2) to investigate the associations among these variables; and (3) to test whether perceived stress mediates the relationship between professional role and trait anxiety (Figure 1).

## Materials and methods

### Participants

The sample consisted of 177 sport professionals, including 94 referees and 83 coaches, recruited from football teams. Participants were selected using a random sampling procedure across different teams to ensure variability in experience and team context. All participants were actively involved in their respective roles during the data collection period and were competing at the regional level. On average, participants had approximately 5 years of experience in their current role. The mean age of the sample was 33.6 years (SD = 2.20), ranging from 30 to 37 years. Participants were regularly involved in regional competitions that typically include regular seasonal schedules with multiple matches per year, reflecting consistent exposure to performance-related demands. Although demographic variables such as gender and years of experience were not included as control variables in the main analyses, participants were recruited to ensure a relatively homogeneous sample in terms of age and competitive level. Participants were eligible for inclusion if they met the following criteria: (a) being currently active as a football referee or coach at the professional level within regional competitions; (b) providing informed consent to participate in the study. Participants were excluded if they: (a) were not actively involved in competitions during the data collection period; (b) provided incomplete or invalid questionnaire responses.

### Procedure

Participants were recruited through direct contact with football teams and officiating organizations at the regional level. Data collection took place shortly before the start of the competitive season, a period characterized by anticipatory demands and preparation for upcoming matches. The questionnaires were administered in a standardized format and completed individually in a quiet setting, either in person or under supervised conditions, to minimize distractions and ensure data quality. The order of questionnaire administration was kept consistent across participants to ensure comparability. Completion time was approximately 15–20 min. To reduce potential response bias, participants were instructed to answer honestly and were assured that there were no right or wrong answers. The anonymity of responses was emphasized to minimize social desirability effects.

Prior to the beginning of the study, all participants were informed about the procedures of the study and the anonymity of their answers before providing their written consent to participate. This study was carried out in accordance with the recommendations of the Ethical Code of the Code of Ethics approved by the General Assembly of the Italian Association of Psychology held on March 27, 2015. The study protocol received official clearance from the Departmental Research Committee (approval number: 822/2025/MEDF-03/10), in accordance with the Declaration of Helsinki.

### Measures

#### Perceived stress

Perceived stress was assessed using the Psychological Stress Measure (PSM-9; Lemyre and Lalande-Markon, 2009), a 9-item self-administered questionnaire measuring directly the state of perceived stress. Items are rated on an 8-point Likert scale (1 = not at all, 8 = extremely). Items include: I feel calm, and I feel full of energy and keen. Positively worded items were reverse-coded, and a total score was computed by summing all items, with higher scores indicating higher levels of perceived stress. The scale has demonstrated high internal consistency (Cronbach’s *α* ≈ 0.92–0.93). The internal consistency for the current study is good (*α* = 0.89).

#### Personality traits

Personality traits were assessed using a short 10-item version of the Big Five Inventory (Gosling et al., 2003). The scale measures five personality dimensions: Extraversion, which is inherent in a confident and enthusiastic orientation toward the various circumstances of life, most of which are interpersonal; Agreeableness, which includes, on the one hand, features such as altruism, caring, giving emotional support, and, on the other, features such as hostility, indifference to others and selfishness; Conscientiousness, which refers to features such as accuracy, reliability, responsibility and perseverance; Emotional stability, which is a very large dimension comprising a variety of features related to anxiety and emotional problems such as depression, mood instability and irritability; Openness, which refers to an openness to new ideas, to the values of others and to one’s own feelings. Given the brevity of the scale, lower internal consistency coefficients are expected, as each dimension is assessed with a limited number of items. However, the instrument has demonstrated adequate validity and has been widely used in research settings, particularly when shorter assessments are required.

#### Anxiety

Anxiety was assessed using the State–Trait Anxiety Inventory (STAI-Y; Spielberger, 1983). The instrument consists of two 20-item subscales measuring state anxiety, referring to the psychophysiological condition of a given moment, and trait anxiety, understood as a personality trait.

Items are rated on a 4-point Likert scale, with total scores ranging from 20 to 80. Higher scores indicate higher levels of anxiety. Internal consistency coefficients for the scale have ranged from 0.86 to 0.95, and test–retest reliability coefficients have ranged from 0.65 to 0.75 over a 2-month interval. The internal consistency measured for our data was good, showing a *α* = 0.86.

### Statistical analyses

Statistical analyses were processed with SPSS version 27.0 (IBM, Armonk, NY, United States). Preliminary analyses included descriptive statistics and frequency distributions to characterize the sample. Assumptions of normality were assessed using the Shapiro–Wilk test, and homogeneity of variances was evaluated using Levene’s test.

To examine differences between coaches and referees, independent samples *t*-tests were conducted for all study variables. When the assumption of homogeneity of variances was violated, Welch’s *t*-test was considered. Effect sizes were reported using Cohen’s *d*. In addition, non-parametric Mann–Whitney *U* tests were performed to confirm the robustness of the results.

To examine group differences across multiple dependent variables simultaneously, a multivariate analysis of variance (MANOVA) was performed, with professional role as the independent variable and personality traits, perceived stress, and anxiety variables as dependent variables. Multivariate effects were assessed using Pillai’s Trace, and effect sizes were reported as partial eta squared (*η*^2^*p*). Pearson’s correlation coefficients were computed to examine associations among personality traits, perceived stress, and anxiety variables.

To investigate predictors of anxiety, hierarchical multiple regression analyses were conducted separately for state anxiety and trait anxiety. In each model, variables were entered in three steps: (1) professional role (coach vs. referee), (2) personality traits (Big Five), and (3) perceived stress. Changes in explained variance (Δ*R*^2^) were examined at each step.

Finally, a mediation analysis was performed using Hayes’ (2013) PROCESS version 3.1 (Hayes PROCESS macro-Model 4—Ohio, United States) to test whether perceived stress mediated the relationship between professional role and trait anxiety. Professional role was entered as the independent variable, perceived stress as the mediator, and trait anxiety as the outcome variable. Indirect effects were estimated using a bootstrapping procedure with 5,000 resamples, and mediation was considered significant when the 95% confidence interval did not include zero (Preacher and Kelley, 2011).

The significance level was set at *p* < 0.05 for all analyses.

## Results

### Preliminary analyses and descriptive statistics

Preliminary analyses were conducted to examine the distribution of the variables and to verify the assumptions for parametric testing. Descriptive statistics indicated that the sample consisted of 177 participants, including 94 referees and 83 coaches, with a mean age of 33.6 years (SD = 2.20). Shapiro–Wilk tests showed deviations from normality for most study variables, with the exception of perceived stress. However, given the sample size and the robustness of parametric procedures, parametric analyses were retained. Homogeneity of variances was supported for most variables, although Levene’s test was significant for conscientiousness and emotional stability, indicating unequal variances for these variables. In these cases, Welch’s *t*-test was also considered. Box’s test of equality of covariance matrices was non-significant, Box’s *M* = 47.482, *F* = 1.255, *p* = 0.141, supporting the assumption of homogeneity of covariance matrices for the MANOVA across groups. Descriptive statistics by professional role are reported in Table 1. Referees showed higher mean scores in trait anxiety and perceived stress, whereas coaches showed higher mean scores in openness, agreeableness, conscientiousness, and emotional stability.

**Table 1:** Mean ± SD for the study variables.

Variables		Role	
		Referee *n* = 94	Coach *n* = 83
Extraversion	*M*	3.64	3.60
	SD	0.82	0.73
Openness	*M*	2.89	3.19
	SD	0.73	0.83
Agreeableness	*M*	2.97	3.39
	SD	0.73	0.74
Conscientiousness	*M*	3.24	3.93
	SD	0.72	0.81
Emotional stability	*M*	2.95	3.16
	SD	0.68	0.89
State anxiety	*M*	30.59	32.33
	SD	8.54	9.73
Trait anxiety	*M*	37.52	33.10
	SD	8.65	10.35
Perceived stress	*M*	47.03	43.08
	SD	6.85	7.80

### Group differences

To examine group differences across multiple dependent variables simultaneously, a multivariate analysis of variance (MANOVA) was performed with professional role as the independent variable and personality traits, anxiety variables, and perceived stress as dependent variables. The MANOVA revealed a significant multivariate effect of professional role, Pillai’s Trace = 0.310, *F* (8, 168) = 9.457, *p* < 0.001, *η*^2^*p* = 0.310, indicating that referees and coaches differed across the combined set of dependent variables.

Follow-up univariate analyses showed significant effects of professional role on openness, *F* (1, 175) = 6.579, *p* = 0.011, *η*^2^*p* = 0.036, agreeableness, *F* (1, 175) = 14.728, *p* < 0.001, *η*^2^*p* = 0.078, conscientiousness, *F* (1, 175) = 34.960, *p* < 0.001, *η*^2^*p* = 0.167, trait anxiety, *F* (1, 175) = 9.592, *p* = 0.002, *η*^2^*p* = 0.052, and perceived stress, *F* (1, 175) = 12.849, *p* < 0.001, *η*^2^*p* = 0.068. No significant differences emerged for extraversion, emotional stability, or state anxiety.

To further examine these group differences, independent samples *t*-tests were conducted. Referees reported significantly higher levels of trait anxiety (*M* = 37.52, SD = 8.65) than coaches (*M* = 33.10, SD = 10.35), *t* (175) = 3.097, *p* = 0.002, *d* = 0.47. Referees also reported significantly higher levels of perceived stress (*M* = 47.03, SD = 6.85) than coaches (*M* = 43.08, SD = 7.80), *t* (175) = 3.585, *p* < 0.001, *d* = 0.54.

Conversely, coaches scored higher than referees on openness (*M* = 3.19 vs. 2.89), *t* (175) = −2.565, *p* = 0.011, *d* = −0.39, agreeableness (*M* = 3.39 vs. 2.97), *t* (175) = −3.838, *p* < 0.001, *d* = −0.58, and conscientiousness (*M* = 3.93 vs. 3.24), *t* (175) = −5.913, *p* < 0.001, *d* = −0.89. No significant differences were found for extraversion, emotional stability, or state anxiety. Mann–Whitney *U* tests yielded the same overall pattern of results, supporting the robustness of the findings.

### Correlations

Pearson correlation coefficients were computed to examine associations among personality traits, perceived stress, and anxiety variables. As shown in Table 2, perceived stress was positively associated with both state anxiety, *r* = 0.225, *p* = 0.003, and trait anxiety, *r* = 0.284, *p* < 0.001. State and trait anxiety were also positively correlated, *r* = 0.306, *p* < 0.001.

**Table 2:** Pearson’s correlation coefficients.

Variables	1	2	3	4	5	6	7	8
1. Extraversion	1							
2. Openness	0.236*							
3. Agreeableness	0.232*	0.059						
4. Conscientiousness	0.267**	0.265**	0.313**					
5. Stability	0.352**	0.233*	0.167	0.376**				
6. State anxiety	0.054	0.109	−0.034	0.094	0.165			
7. Trait anxiety	−0.028	−0.118	−0.163	−0.234*	−0.021	0.306**		
8. Perceived stress	0.156	−0.091	−0.045	0.028	0.240**	0.225*	0.284**	1

*p < 0.05; **p < 0.001.

Among personality traits, agreeableness was negatively associated with trait anxiety, *r* = −0.163, *p* = 0.030, and conscientiousness was also negatively associated with trait anxiety, *r* = −0.234, *p* = 0.002. Emotional stability was positively associated with perceived stress, *r* = 0.240, *p* = 0.001, and state anxiety, *r* = 0.165, *p* = 0.028. Extraversion showed a small positive association with perceived stress, *r* = 0.156, *p* = 0.038.

### Hierarchical regression analyses

#### State anxiety

A hierarchical multiple regression was conducted to examine predictors of state anxiety. In Step 1, professional role did not significantly predict state anxiety, *R*^2^ = 0.009, *F* (1, 175) = 1.605, *p* = 0.207. In Step 2, the inclusion of personality traits did not significantly improve the model, Δ*R*^2^ = 0.034, *F* change (5, 170) = 1.226, *p* = 0.299. In Step 3, the inclusion of perceived stress produced a significant increase in explained variance, Δ*R*^2^ = 0.053, *F* change (1, 169) = 9.988, *p* = 0.002. The final model was significant, *R*^2^ = 0.097, adjusted *R*^2^ = 0.060, *F* (7, 169) = 2.592, *p* = 0.015.

In the final model, perceived stress emerged as the only significant predictor of state anxiety, *β* = 0.253, *p* = 0.002. Professional role and all personality traits were non-significant. Collinearity diagnostics indicated no evidence of problematic multicollinearity (all VIFs <1.50).

#### Trait anxiety

A second hierarchical multiple regression was conducted to examine predictors of trait anxiety. In Step 1, professional role significantly predicted trait anxiety, *R*^2^ = 0.052, *F* (1, 175) = 9.592, *p* = 0.002. In Step 2, the inclusion of personality traits increased the explained variance to 9.0%, Δ*R*^2^ = 0.038, but this change was not statistically significant, *F* change (5, 170) = 1.427, *p* = 0.217. In Step 3, the inclusion of perceived stress significantly improved the model, Δ*R*^2^ = 0.058, *F* change (1, 169) = 11.605, *p* = 0.001. The final model was significant, *R*^2^ = 0.149, adjusted *R*^2^ = 0.113, *F* (7, 169) = 4.214, *p* < 0.001.

In the final model, perceived stress was a significant positive predictor of trait anxiety, *β* = 0.265, *p* = 0.001, whereas conscientiousness was a significant negative predictor, *β* = −0.193, *p* = 0.028. Professional role was no longer significant after the inclusion of personality traits and perceived stress, suggesting that its initial effect was accounted for by these variables. No other personality traits emerged as significant predictors. Collinearity diagnostics again indicated no multicollinearity concerns.

### Mediation analysis

To further examine whether perceived stress explained the association between professional role and trait anxiety, a mediation analysis was conducted with professional role as the predictor, perceived stress as the mediator, and trait anxiety as the outcome variable.

The analysis showed that professional role significantly predicted perceived stress, *β* = −3.948, SE = 1.114, 95% CI [−6.216, −1.908], *p* < 0.001. In turn, perceived stress significantly predicted trait anxiety, *β* = 0.309, SE = 0.086, 95% CI [0.144, 0.490], *p* < 0.001.

The indirect effect of professional role on trait anxiety through perceived stress was significant, *β* = −1.22, SE = 0.492, 95% CI [−2.37, −0.42], *p* = 0.013, indicating that perceived stress mediated the relationship between role and trait anxiety.

The direct effect of professional role on trait anxiety remained significant after including the mediator, *β* = −3.20, SE = 1.415, 95% CI [−5.98, −0.23], *p* = 0.024, suggesting a partial mediation. The total effect of professional role on trait anxiety was also significant, *β* = −4.42, SE = 1.445, 95% CI [−7.29, −1.69], *p* = 0.002.

Overall, these findings are consistent with the hypothesis that perceived stress partially accounts for the association between professional role and trait anxiety.

## Discussion

The present study adopted a theory-informed explanatory framework to investigate the psychological processes underlying anxiety in sport professionals. Specifically, it examined whether perceived stress could represent a potential explanatory pathway linking professional role and trait anxiety in football coaches and referees.

### From group differences to potential explanatory pathways

Consistent with previous research, referees reported higher levels of perceived stress and trait anxiety compared to coaches. While such differences have been documented in previous research, the present findings extend this literature by moving beyond descriptive comparisons and identifying a potential explanatory pathway associated with these differences. From a theoretical standpoint, these results support the idea that psychological outcomes in sport contexts are not solely determined by role-specific demands, but by how these demands are cognitively appraised. In line with transactional models of stress (Biggs et al., 2017), the findings are consistent with the possibility that referees’ greater exposure to time pressure, evaluation, and decision-making uncertainty may be associated with higher levels of perceived stress and trait anxiety.

### Perceived stress as a potential explanatory process

The main contribution of this study lies in providing empirical support for a theory-informed explanatory framework by identifying perceived stress as a potential explanatory pathway linking professional role and trait anxiety in football professionals. Perceived stress was consistently associated with both state and trait anxiety and emerged as the strongest predictor in the regression models.

Most notably, the mediation analysis indicated that perceived stress partially mediated the relationship between professional role and trait anxiety.

These findings suggest that differences in anxiety between coaches and referees may be partly associated with differences in how stressful those roles are perceived to be.

These results align with theoretical perspectives emphasizing the role of cognitive appraisal in emotional responses and extend previous work by providing empirical evidence of a mediational process in sport professionals (Lavoie et al., 2021). The present findings are consistent with previous research highlighting the central role of stress in referees’ psychological functioning and its close association with anxiety and performance-related outcomes. In line with recent theoretical and empirical work, the results suggest that stress responses in sport are not determined solely by objective demands, but by how these demands are cognitively appraised. Perceived stress appears to represent a potential explanatory pathway linking contextual demands to emotional outcomes, consistent with transactional models of stress (Aguirre-Loaiza et al., 2020; Robazza et al., 2023). At the same time, alternative interpretations should be considered. Individuals with higher levels of trait anxiety may be more likely to perceive situations as stressful, suggesting that the relationship between perceived stress and trait anxiety could be reciprocal. Because the present study employed a cross-sectional design, the temporal ordering of these variables cannot be established. Therefore, the findings should be interpreted as being consistent with the proposed theoretical framework rather than as evidence of a directional or causal relationship.

### The role of personality: a secondary but meaningful contribution

Although personality traits were not the primary focus of the study, the findings regarding conscientiousness provide additional insight. Conscientiousness emerged as a significant negative predictor of trait anxiety, suggesting a protective role.

From a theoretical perspective, individuals high in conscientiousness may possess greater self-regulatory capacity, planning skills, and behavioral control, which may reduce vulnerability to anxiety in demanding environments. This finding supports previous literature linking conscientiousness to adaptive coping and lower psychological distress (Vollrath (2001).

However, personality traits did not account for the differences between coaches and referees, nor did they overshadow the role of perceived stress. This suggests that situational appraisal processes may be more proximal determinants of anxiety than dispositional traits in this context (Bouchard et al., 2004). These findings should, however, be interpreted with caution because personality traits were assessed using a brief two-item measure for each dimension. Although the instrument has demonstrated acceptable validity for research purposes, its limited length may have reduced measurement precision compared with more comprehensive personality inventories.

### Partial mediation and the role of additional factors

Importantly, the mediation was partial, as the direct effect of professional role on trait anxiety remained significant. This indicates that perceived stress partially accounts for the observed association between professional role and trait anxiety.

This finding suggests that other factors may contribute to the observed differences in anxiety.

Potential candidates include coping strategies, emotional regulation, perceived competence, and exposure to social evaluation (Hanin et al., 2021; Liem, 2022). Future research should explore these variables to develop a more comprehensive model of psychological functioning in sport professionals.

### Implications for theory and practice

From a theoretical perspective, the present study does not propose a new theoretical model but provides empirical support for transactional models of stress within the context of football. Its primary contribution lies in applying a theory-informed explanatory framework to simultaneously examine professional role, personality traits, perceived stress, and anxiety within a single analytical framework. In this way, the study extends previous research by moving beyond descriptive comparisons between coaches and referees and by evaluating whether perceived stress represents a potential explanatory pathway linking professional role and trait anxiety (Yeo and Ong, 2024).

From an applied perspective, the findings suggest that perceived stress may represent a promising target for interventions aimed at supporting the psychological well-being of sport professionals, particularly referees. Approaches focusing on stress appraisal, coping strategies, and cognitive restructuring may represent promising intervention targets; however, their effectiveness cannot be inferred from the present findings and should be evaluated in future longitudinal and intervention-based studies. Likewise, strategies aimed at strengthening self-regulatory skills, including those related to conscientiousness, may deserve further investigation as potential approaches for promoting resilience to stress and anxiety. However, these theoretical implications should be interpreted within the context of the present sample, consisting of male football coaches and referees competing at the regional level. Further research is needed to determine whether similar patterns emerge in other sports and competitive contexts.

### Limitations and future directions

Despite these contributions, several limitations should be acknowledged. First, the present study focused exclusively on football, a sport characterized by high cognitive demands, rapid decision-making, and strong social evaluation, particularly for referees. This choice allowed for greater control over sport-specific variability and enhanced the internal validity of the findings. However, given that different sports involve distinct performance demands and stressors, the results may not be generalizable to other sport contexts. Future research should examine whether similar psychological mechanisms emerge across different sports. In addition, the sample consisted exclusively of male participants, reflecting the demographic composition of referees and coaches at the regional level. While this limits the generalizability of the findings, it also reduced variability associated with potential gender differences in stress perception and anxiety responses. Future studies should include more diverse samples to further explore the role of gender in these processes. Although the sample was relatively homogeneous in terms of age and competitive level, other demographic characteristics, such as years of experience, gender, and competitive level, may have influenced the observed associations. These variables were not included as covariates in the present analyses and may represent potential sources of variability in perceived stress and anxiety. Future studies should examine their contribution more explicitly, either as control variables or as potential moderators of the relationships observed in the present study.

Furthermore, the cross-sectional design limits causal inference, and the use of self-report measures may introduce bias. Therefore, the mediation model should be interpreted as being consistent with the proposed theoretical framework rather than as evidence of causal relationships. Future longitudinal studies are needed to establish the temporal ordering among professional role, perceived stress, and trait anxiety. Personality traits were assessed using a brief two-item measure for each dimension. Although this instrument has demonstrated adequate validity and is appropriate for research requiring brief assessments, its limited length may have reduced measurement precision. Therefore, the personality findings should be interpreted with appropriate caution. Finally, longitudinal and intervention-based studies would help clarify the causal relationship between perceived stress and anxiety and evaluate the effectiveness of stress-management programs. Moreover, integrating psychological and physiological indicators of stress may provide a more comprehensive understanding of the mechanisms underlying anxiety in sport professionals. Future research should also explore additional mediators and moderators to further refine the proposed theoretical model. These developments could contribute to the design of evidence-based interventions aimed at enhancing psychological well-being and performance in coaches and referees.

## Conclusion

In conclusion, the present study provides empirical support for the view that perceived stress represents a potential explanatory pathway associated with trait anxiety among football professionals. Specifically, differences in trait anxiety between coaches and referees appear to be partly associated with differences in how stressful their professional roles are perceived to be. These findings are consistent with the view that psychological outcomes in sport contexts are influenced not only by objective role demands but also by the way these demands are cognitively appraised.

By adopting a theory-informed explanatory framework, this study extends previous research by highlighting perceived stress as a potential explanatory pathway linking professional role and trait anxiety. The findings are consistent with transactional models of stress, although longitudinal research is needed to establish causal relationships. In doing so, it contributes to a growing body of literature emphasizing the importance of cognitive appraisal processes in understanding stress and anxiety in sport. From an applied perspective, these findings suggest that perceived stress may represent a promising target for interventions aimed at improving psychological well-being in sport professionals, particularly in high-pressure roles such as refereeing.

From an applied perspective, these findings underline the importance of targeting perceived stress in interventions aimed at improving psychological well-being in sport professionals, particularly in high-pressure roles such as refereeing. Overall, the study provides further evidence that understanding how individuals interpret and respond to stressors is essential for advancing both theory and practice in sport psychology.

## Data Availability

The raw data supporting the conclusions of this article will be made available by the authors, without undue reservation.
